# 3-Methylcholanthrene, an AhR Agonist, Caused Cell-Cycle Arrest by Histone Deacetylation through a RhoA-Dependent Recruitment of HDAC1 and pRb2 to E2F1 Complex

**DOI:** 10.1371/journal.pone.0092793

**Published:** 2014-03-21

**Authors:** Chih-Cheng Chang, Yuh-Mou Sue, Nian-Jie Yang, Yi-Hsuan Lee, Shu-Hui Juan

**Affiliations:** 1 Department of Physiology, School of Medicine, College of Medicine, Taipei Medical University, Taipei, Taiwan; 2 Graduate Institute of Medical Sciences, Taipei Medical University, Taipei, Taiwan; 3 Department of Nephrology, Taipei Medical University-Wan Fang Hospital, Taipei, Taiwan; 4 Institute of Physiology, National Yang-Ming University, Taipei, Taiwan; Nihon University School of Medicine, Japan

## Abstract

We previously showed that treating vascular endothelial cells with 3-methylcholanthrene (3MC) caused cell-cycle arrest in the Go/G1 phase; this resulted from the induction of p21 and p27 and a decreased level and activity of the cyclin-dependent kinase, Cdk2. We further investigated the molecular mechanisms that modulate cell-cycle regulatory proteins through the aryl-hydrocarbon receptor (AhR)/Ras homolog gene family, member A (RhoA) dependent epigenetic modification of histone. AhR/RhoA activation mediated by 3MC was essential for the upregulation of retinoblastoma 2 (pRb2) and histone deacetylase 1 (HDAC1), whereas their nuclear translocation was primarily modulated by RhoA activation. The combination of increased phosphatase and tensin homolog (PTEN) activity and decreased phosphatidylinositide 3-kinase (PI3K) activation by 3MC led to the inactivation of the Ras-cRaf pathway, which contributed to pRb2 hypophosphorylation. Increased HDAC1/pRb2 recruitment to the E2F1 complex decreased E2F1-transactivational activity and H3/H4 deacetylation, resulting in the downregulation of cell-cycle regulatory proteins (Cdk2/4 and Cyclin D3/E). Co-immunoprecipitation and electrophoretic mobility shift assay (EMSA) results showed that simvastatin prevented the 3MC-increased binding activities of E2F1 proteins in their promoter regions. Additionally, RhoA inhibitors (statins) reversed the effect of 3MC in inhibiting DNA synthesis by decreasing the nuclear translocation of pRb2/HDAC1, leading to a recovery of the levels of cell-cycle regulatory proteins. In summary, 3MC decreased cell proliferation by the epigenetic modification of histone through an AhR/RhoA-dependent mechanism that can be rescued by statins.

## Introduction

Environmental contamination by polycyclic aromatic hydrocarbons, such as 2,3,7,8-tetrachlorodibenzodioxin, polychlorinated biphenyls, and 3-methylcholanthrene (3MC) adversely affects wildlife and human health. Biochemical and genetic studies have shown that the action of 2,3,7,8-tetrachlorodibenzodioxin (TCDD) and 3MC is mediated by aryl hydrocarbon receptors (AhRs). The AhR is a ligand-activated transcription factor and acts as the receptor for polycyclic aromatic hydrocarbons, polychlorinated biphenyls, and TCDD, which diffuses across plasma membranes and binds to the AhR complex present in the cytoplasmic compartment [1]. Ligand AhR complexes are then translocated into the nucleus, where they interact with the nuclear aryl hydrocarbon receptor nuclear translocator (Arnt) protein. Thereafter, the ligand-AhR-Arnt complex binds to the dioxin responsive element (DRE) on DNA, initiating the transcription of genes, including cytochrome P-450(CYP), FAK, RhoA, p21/p27, integrin, and fibronectin [2]–[5].

The cell cycle is mediated by the activation of cyclins and cyclin-dependent kinases (Cdks), proteins that jointly initiate progression from the G1 phase to the S phase of the cell cycle, and from the G2 phase to mitosis. The cyclins have been identified as cyclins A, D1, D3, and E; and the most common Cdks are Cdk2 and Cdk4. The cyclin A–Cdk2 and cyclin E–Cdk2 complexes form late in the G1 phase, when cells are preparing to synthesize DNA [6] and the formation of cyclin E is a rate-limiting step in the G1/S transition [7]. The cyclin-Cdk complexes are regulated in cell-cycle progression by Cdk inhibitors such as p21/Cip1 and p27/Kip1, which prevent abnormal proliferation by blocking catalytic activity [8]. Our previous study showed that treating human umbilical cord vascular endothelial cells (HUVECs) with 3MC increased the upregulation of p21 and p27 and decreased the level and activity of Cdk2; these alterations resulted in cell-cycle arrest in the G0/G1 phase [4].

E2 transcription factor (E2F) is known to regulate the G1/S phase transition of the cell cycle by modulating the transcription of essential cell-cycle control genes. Hypophosphorylated forms of pRb bind to E2F, resulting in transcriptional repression. Simultaneously, pRb phosphorylation by the cyclin-Cdk complex or the Ras-cRaf pathway releases E2F, which activates transcriptional regulation in proteins regulated by the cell cycle [9], [10]. Ras has to be palmitoylated and consequently membrane-localized in order to efficiently activate a downstream effector, c-Raf [11]. Additionally, c-Raf has been reported to bind physically to pRb in growth-factor-stimulated cells, interacting with only the active (hypophosphorylated) form of pRb and resulting in the increase of pRb phosphorylation and cell-cycle progression [9]. Moreover, the interaction between pRb and chromatin-modifying co-repressors mediates the active repression of E2F-reponsive genes. It has been reported that one member of the pRb family, pRb2/p130, encodes a cell cycle regulatory protein and has been found mutated in various tumors. The overexpression of pRb2/p130 not only suppresses tumor formation in nude mice, but also causes the regression of established tumor grafts, suggesting that pRb2/p130 modulates the angiogenetic balance [12].

Chromatin modification is crucial for gene regulation. For example, histone deacetylation by histone deacetylases (HDACs) condenses the chromatin structure, thereby decreasing the accessibility of transcription factors and resulting in the downregulation of target genes. By contrast, the reverse reaction is catalyzed by histone acetyltransferases, which promote transcriptional activation. Histone remodeling factors, such as the HDAC family and CBP/p300, modulate the AhR-mediated gene regulation [13]. Additionally, the effect of AhR agonists in cell-cycle inhibition has been demonstrated extensively among various cell types [14], [15]. Previous reports have elucidated a direct interaction between the AhR and pRb [16], [17]. The activated AhR, which associates with the hypophosphorylated pRb [16], results in G_1_ arrest [17], [18]. The negative regulation has also been shown in the physical interaction between AhR and pRb [19]. HDAC1 is essential for repressing Cyclin E gene expression by recruitment to pRb-E2F complexes [20]. Nevertheless, the involvement of AhR in epigenetic modification and its underlying molecular mechanisms in regulating the cell cycle remain unclear.

The Rho family of GTPases (including Rho, Rac, and Cdc42) has been identified as signaling molecules that regulate cytoskeletal rearrangement and are involved in cellular functions such as smooth muscle cell contraction and cell migration. Rac and Cdc42 respectively regulate lamellipodia and filopodia formation at the leading edge of a migrating cell, and Rho enables the formation and maintenance of focal adhesions [21]. RhoA is widely considered a regulator of F-actin stress fiber formation. We previously showed that AhR not only upregulated RhoA levels through a genomic pathway, but also increased RhoA activity by blocking the negative feedback of FAK/p190RhoGAP. Additionally, RhoA activation results in stress fiber formation and the anti-migratory effect of 3MC in HUVECs [3]. A recent study showed that RhoA enhances the nuclear translocation of NF-κB, thereby affecting the transcription regulation of inflammatory genes in cells exposed to a thrombin challenge [22]. However, further investigation is required to determine whether RhoA activation is associated with an increased nuclear translocation of other transcription factors in cell-cycle regulation.

In considering our previous finding of cell-cycle arrest in the G0/G1 phase by 3MC, we explored the hypothesis that AhR/RhoA activation involves epigenetic modification of chromatin, which in turn inhibits cell-cycle regulatory genes and leads to reduced DNA incorporation. Based on the molecular mechanisms underlying the action of 3MC, we investigated whether RhoA inhibitors (simvastatin and pravastatin) can protect vascular endothelial cells from AhR-mediated anti-proliferation.

## Materials and Methods

### Mouse cerebral vascular endothelial cells (MCVECs) primary cultures and reagents

The MCVECs were prepared as described previously [23]. All procedures were conducted in accordance with the Taipei medical university animal care and use rules (licenses No. LAC-99-0205) and an Association for Assessment and Accreditation of Laboratory Animal Care approved protocol. The MCVECs were obtained from 6 male Balb/c mice (aged 3–4 wk). The animals were anesthetized intramuscularly with a combination of ketamine (8 mg/100 g body weight) and xylazine (2 mg/100 g). Briefly, the mice were perfused and decapitated. After removing the meninges, white matter, and superficial blood vessels, the mice cortices were minced and gently dissociated in an ice-cold Hank's balanced salt solution (GIBCO, Grand Island, NY). The gray matter was homogenized and filtered, and the resulting microvessel fraction was then digested with collagenase/dispase at a concentration of 1 mg/mL (Sigma-Aldrich, St. Louis, MO) for 6 h at room temperature. After centrifugation, the pellet containing the MCVECs was washed with Dulbecco's Modified Eagle's Medium (DMEM, GIBCO), and maintained in the DMEM supplemented with 10% fetal bovine serum (FBS) in a humidified incubator (37°C, 5% CO_2_). The cells were stained with CD31 for the identification of vascular endothelial cells. Cells from passages 10 to 25 were used. The DMEM, FBS, and tissue culture reagents were obtained from Life Technologies (Gaithersburg, MD). Additional reagents were purchased from the following sources: 3MC from Supelco (Bellefonte, PA); YS-49 (a PI3K activator), simvastatin, and pravastatin from Sigma-Aldrich (St. Louis, MO); Y27632 from Calbiochem (San Diego, CA); N-[5-(2-(2,6-dichloro-phenyl)-5-difluoromethyl-2H-pyrazol-3-yl)-thiazol-2-yl] (bpV[phen]; a PTEN inhibitor) from Santa Cruz Biotechnology (Santa Cruz, CA); and BMS-5 (a LIM kinase [LIMK] inhibitor) from Activate Scientific GmbH (Prien, Germany).

### Transfection of AhR/HDAC1 small interfering (si)RNAs and RhoA/Ras variants, and reverse-transcription polymerase chain reaction (RT-PCR) analysis

AhR siRNA (UUACUAUCUUGAAAGAGCCct) and HDAC1 siRNA (AGUGCUGUGAAGCUUAAUATT) duplexes were chemically synthesized using Ambion (Austin, TX) and MDBio, Inc. (Taipei, Taiwan), respectively. The MCVECs were seeded in 6-well plates and transfected with 5 pmol of AhR siRNA or 20 pmol of HDAC1 siRNA in a 100 μL volume with a TransIT-TKO transfection reagent (Mirus Bio Corporation, Madison, WI). The RhoA complementary (c) DNAs (T19N dominant negative [DN] and Q63L constitutive active [CA]) in pUSEamp were purchased from Millipore (Burlington, MA), and dominant negative H-Ras (S17N) was obtained from Upstate Biotechnology (Lake Placid, NY). We transfected the pUSEamp-overexpressing variants (4 μg/3.5 cm Petri dish) into the MCVECs by using the jetPEI™ system (Polyplus-transfection, San Marcos, CA). After transfection, cells were plated in DMEM with a 10% FBS.

We used the method described previously [24] to obtain total RNA for the RT-PCR analyses, with minor modifications. Sequences of the primer pairs for amplification of each gene were 5′-CATTCCTCTTCCCCTCATCA-3′ and 5′-GCAGCCCAGAAGAATTTCAG-3′ (for the Cdk2 gene; 238 bp); 5′-CAATGTTGTACGGCTGATGG-3′ and 5′-CAGGCCGCTTAGAAACTGAC-3′ (for the Cdk4 gene; 178 bp); 5′-AGACCTTTTTGGCCCTCTGT-3′ and 5′-GTCCACTTCAGTGCCTGTGA-3′ (for the cyclin D3 gene; 176 bp); 5′-CCTCCAAAGTTGCACCAGTT-3′ and 5′-GGACGCACAGGTCTAGAAGC-3′ (for the cyclin E gene; 241 bp); and 5′-CCACCATGGAGAAGGCTGGGGCTCA-3′ and 5′-ATCACGCCACAGTTTCCCGGAGGGG-3′ (for the GAPDH gene).

### Preparation of cell fractions (nuclear, cytosolic, and membrane) and western blot analysis

The MCVECs were harvested in 10-cm^2^ dishes after the indicated treatment. The cells were partitioned into cytosolic and nuclear fractions using NE-PER™ nuclear extraction reagents (Pierce, Rockford, IL) with the addition of protease inhibitors, according to manufacturer instructions. To prepare membrane-cytosolic fractions, after the indicated treatment, the cells were collected and incubated in 0.1 mL of hypotonic buffer (10 mM of Tris [pH 7.5], 0.5 mM of EDTA, and 2 mM of phenylmethylsulfonyl fluoride) at 4°C for 30 min. After centrifugation, the supernatant (cytosolic fraction) was collected, and the pellet was resuspended in 0.1 mL of radioimmunoprecipitation assay buffer, and then incubated at 4°C for 30 min. The resulting fractions were sheared 100 times through an insulin syringe using a 29-gauge needle. After centrifugation, the supernatant (membrane fraction) was collected for analysis. The cell lysates (30 μg) were electrophoresed on a 10% sodium dodecylsulfate (SDS)-polyacrylamide gel and then transblotted onto a Hybond-P membrane (GE Healthcare, Hong Kong).

The assay included antibodies for the following: pc-Raf (Cell Signaling Technology, Beverly, MA); Histone H3/H4, pRb2, E2F1, H-Ras, α-tubulin (Santa Cruz Biotechnology); cyclin D3/E, Cdk2/4, pRb2 (BD BioSciences PharMingen, San Diego, CA); HDAC1 (Abcam, Cambridge); VE-cadherin (Sigma-Aldrich, St. Louis, MO, USA); pPTEN, c-Raf (Epitomics, Burlingame, CA); and GAPDH (Biogenesis, Kingston, NH). The cell lysate (50 μg) was electrophoresed on an 8% SDS-polyacrylamide gel and was then transblotted onto a Hybond-P membrane (GE Healthcare). The following procedures were previously described [24].

### Constructs of the E2 transcription factor (E2F) and DRE enhancers and luciferase activity assay

A luciferase reporter plasmid driven by the E2F was prepared for the assay. In brief, the oligonucleotide with a quadruple repeat of the E2F obtained from the human B-myb promoter (CTTGGCGGGAGA) was cloned into the KpnI and BglII sites of the pGL2-promoter vector (Promega, Madison, WI), and was designated pGL2-E2F [25]. Similarly, the oligonucleotide with a triple repeat of the DRE obtained from the rat CYP1A1 enhancer region (GAGTTGCGTGAGAAGAGCC) was cloned into the pGL2-promoter vector and designated pGL2-3DRE, as described previously [4]. For the reporter activity assay, cells were seeded in 24-well plates at a density of 5×10^4^ cells/well. Cells in each well were transiently transfected with 0.55 μg of plasmid DNA containing 0.05 μg of the Renilla luciferase construct pRL-TK as an internal control (Promega), and 0.5 μg of pGL2-E2F promoter firefly luciferase. We used the jetPEI™ system (Polyplus-Transfection, San Marcos, CA) to prepare the MCVECs. After transfection (4 h), the medium was replaced with fresh medium supplemented with 10% FBS, and incubation continued for another 20 h. Luciferase activities were recorded using a TD-20/20 luminometer (Turner Designs, Madison, WI) with a dual luciferase assay kit (Promega), according to manufacturer instructions. The experimental reporter luciferase activity was calculated by normalizing the intrinsic activity measured for samples corresponding to the pGL2-promoter, and the value to obtain the transfection efficiency as measured according to the activity derived from pRL-TK.

### Co-immunoprecipitation

The pRb2, E2F1, c-Raf, or HDAC1 was immunoprecipitated from 200 μg of protein by using anti-pRb2 or anti-E2F1 antibodies (2 μg) and protein A and G agarose beads (20 μg). The precipitates were washed 5 times with a lysis buffer and once with PBS. The pellet was then resuspended in the sample buffer (50 mM of Tris, [pH 6.8], 100 mM of bromophenol blue, and 10% glycerol) and incubated at 90°C for 10 min before electrophoresis, to release the proteins from the beads.

### Electrophoretic mobility shift assay (EMSA) and chromatin immunoprecipitation (ChIP) assay

The EMSA was performed as described in [26], with minor modifications. To prepare the nuclear protein extracts, MCVECs in 10-cm^2^ dishes were treated with 100 nM of 3MC for 1 h after 1 h of simvastatin pretreatment and then subjected to NE-PER™ nuclear extraction reagents (Pierce, Rockford, IL) with added protease inhibitors. Subsequent procedures for the nuclear protein extraction involved following manufacturer instructions. Sequences of the oligonucleotides used were as follows: 5′-GGAACTGCGGGAAAGTTGTG-3′ wild-type (WT) and 5′-GGCCCTTATTGCCCGTTTTG-3′ mutant (mut) for the E2F-responsive element (RE) WT and mutant of Cdk2; 5′-CCAATGGCGGGAAGTGGGGC-3′ WT and 5′-ACCATTTATGTCCGTGGTTC-3′ mut for the E2F-RE WT and mutant of Cdk4; 5′-TTCCTCTTTCCTGCCTTCCT-3′WT and 5′-TTCAGATGGACGTAATTACT-3′mut for the putative E2F-RE WT and a mutant of Cyclin D3; and 5′-CTCAGGGGCGGGGAGGACGA-3′ WT and 5′-CTCCGGTTATGTTCGTCAGA-3′ mut for the putative E2F-RE WT and mutant of Cyclin E, respectively. (The conserved and mutated sequences are shown with single and double underlining, respectively).

The oligonucleotides were end-labeled with biotin according to manufacturer protocol (Pierce Biotechnology, Rockford, IL). In brief, unlabeled oligonucleotides (1 μM) were incubated in a TdT reaction buffer containing biotin-11-dUTP (0.5 μM) and TdT (0.2 U/μL) at 37°C for 30 min. This was followed by adding 2.5 μL of EDTA (0.2 M, pH 8.0) to stop each reaction, and the addition of 50 μL of chloroform/isoamyl alcohol to extract the TdT. The extracted nuclear proteins (10 μg) were incubated with biotin-labeled (1 pmol) probes at 15°C for 30 min in a binding buffer containing 1 μg of poly deoxyinosinedeoxycytidine (Panomics, Redwood City, CA). For competition with labeled oligonucleotides, a 100-fold molar excess of unlabeled oligonucleotides relative to biotin-labeled probes was added to the binding assay. The mixture was separated on a 6% nondenaturing polyacrylamide gel at 4°C in 1x TBE (90 mM of Tris borate and 2 mM of EDTA; pH 8.3) and was then transblotted onto a Hybond N^+^ membrane (Amersham Pharmacia Biotech, Freiburg, Germany). Blots were incubated with a blocking buffer, followed by additional streptavidin-horseradish peroxidase conjugates. Blots were imaged using an enhanced chemiluminescence system.

A ChIP assay was performed according to the instructions of Upstate Biotechnology (Lake Placid, NY) with minor modifications. In brief, 6×10^5^ cells that were cultured in 10-cm^2^ dishes with the indicated treatments were harvested. The resulting supernatant was subjected to overnight co-immunoprecipitation (co-IP) using either an anti-E2F1 or anti-pRb2 antibody. The DNA filtrates were amplified by performing a PCR with primers flanking the promoter of Cyclins D3 and E, Cdk2, and 4 genes containing the putative HDAC1/pRb2-E2F1-binding sites. These were Cdk2 forward primer 5′-GGGGTGGTGAAAATTGTGAG-3′ and reverse primer 5′-TCCGATTGGTTCACGTCAC-3′; Cdk4 forward primer 5′-TAGCCGAGCGTAAGGTGAGT-3′ and reverse primer 5′-CATCGCACTAGGCACAAAGA-3′; Cyclin D3 forward primer 5′-GGCTGAGCAGAGAGAGCAGT-3′ and reverse primer 5′-TGAAGACAGAGGCAAGCAGA-3′; and Cyclin E forward primer 5′-GAATGGGTGGATGAATGGAC-3′ and reverse primer 5′-CGGAGTTAAGAACCCGTCAT-3′. In addition, the template was replaced with double-distilled H_2_O as a negative internal control. The PCR products were electrophoresed on a 2% agarose gel; products of the expected sizes of 193 bp, 285 bp, 208 bp, and 245 bp were visualized and quantified using an image analysis system.

### 5-bromo-2-deoxyuridine (BrdU) incorporation and immunofluorescence

The MCVECs were pretreated with simvastatin or pravastatin for 1 h; thereafter, they were subjected to 16 h of a 3MC challenge. Prior to harvesting, the cells were pulsed with BrdU (Invitrogen; 0.75 μg/mL) for 15 h. The cells were fixed in 3.7% formaldehyde and permeabilized with 0.2% Triton X-100 in phosphate-buffered saline. The cells were stained with a mouse anti-BrdU antibody-conjugated with a Texas Red (Jackson ImmunoResearch Laboratories, West Grove, PA). The DNA was stained with 4′, 6-diamidino-2-phenylindole (DAPI, Invitrogen). The red-positive cells represented the BrdU incorporation. Fluorescence was viewed using a CCD camera (DP72, Olympus, Melville, NY) attached to a microscope system (BX51, Olympus) at 200× magnification. Four coverslips were examined in each experimental group.

### Statistical analysis

Values are expressed as the mean ± the standard error of the mean (SEM) for data from at least 3 experiments. The p-values for the differences in means between experimental and control groups were calculated using the Student's t-test or a one-way ANOVA with the Bonferroni method applied as a post hoc test. A value of P<0.05 was considered statistically significant.

## Results

### Alterations in Ras/pc-Raf/pRb2/HDAC1 in relation to the decreased levels of cell-cycle regulatory proteins by 3MC

**Figure 1:**
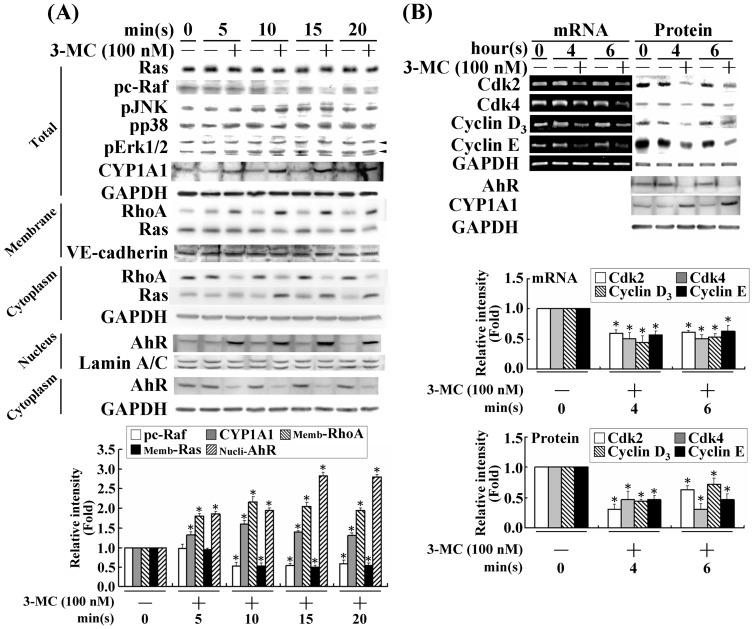
3MC-mediated inactivation of Ras and c-Raf in relation to suppression of Cdk2/4 and Cyclin D3/E1 expression in MCVECs. (A) Cells were transiently challenged by 3MC (0 to 20 min) and then active forms of AhR, RhoA, Ras, c-Raf, and the MAPK family were analyzed using Western blot analysis of the indicated subcellular fractions. Anti-GAPDH, anti-Lamin A/C, and anti-VE-cadherin antibodies were used to verify the equivalent loading amounts of the cytosolic, nuclear, and membrane fractions, respectively. (B) Cells were treated with 3MC for a prolonged period (4 or 6 h) to investigate the effect of 3MC on cell-cycle regulatory proteins, including Cdk2/4 and Cyclin D3/E with CYP1A1 as a positive control for the AhR action of 3MC in Western blot analyses. Total RNA was extracted and analyzed, and GAPDH was used as an internal control. The data are presented as mean ± SEM for 3 independent experiments (^*^ *P*<0.05 vs. control group).

**Figure 2:**
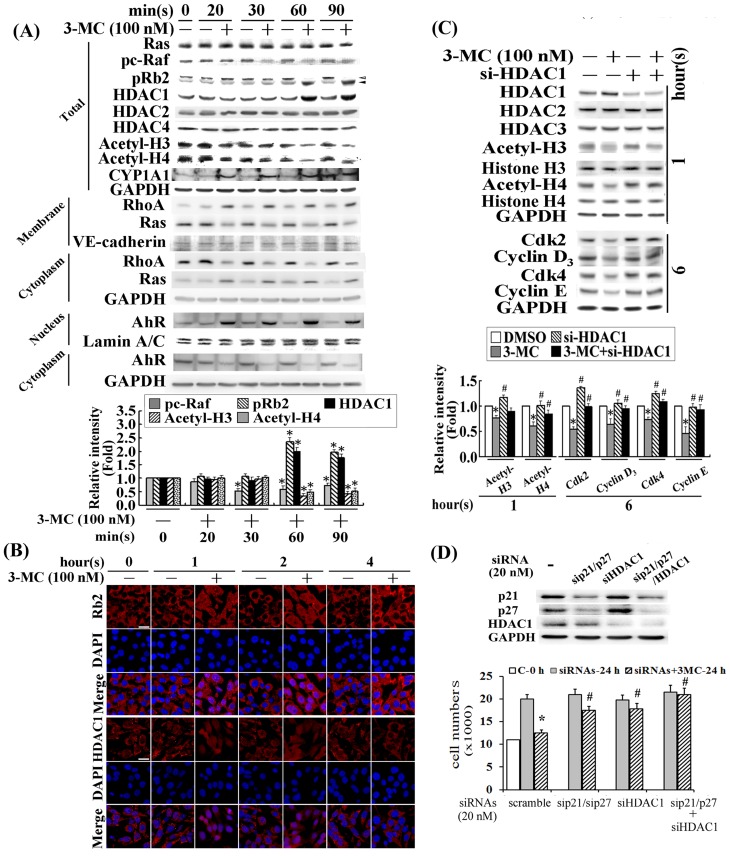
Effects of HDAC1/pRb2 nuclear translocation induced by 3MC in the downregulation of cell-cycle regulatory proteins by histone deacetylation. (A) Cells were harvested and fractionated from MCVECs treated with 100 nM of 3MC at the indicated times, and were analyzed using Western blot. Anti-GAPDH, anti-Lamin A/C, and anti-VE-cadherin antibodies were used to verify the equivalent loading amounts of the cytosolic, nuclear, and membrane fractions, respectively. The open and closed arrowheads represented the hyper and hypophosphorylated pRb2, respectively. (B) The effect of 3MC on RB2 and HDAC1 subcellular localization was investigated using immunofluorescent chemical staining. Cells cultured in coverslips were challenged by 3MC for 1 h; this was followed by fixation and hybridization using anti-pRb2 and anti-HDAC1 antibodies and then a second antibody conjugated with Texas Red. In Figure 2B, red represents pRb2 or HDAC1-positive staining in the cytosol or nuclei. The identical fields were also stained using DAPI to target the nuclear position. We used 630× magnification (scale bar in white  = 50 μm) and recorded micrographs of the representative fields. (C) Cells were transfected with siHDAC1 (20 nM) overnight, prior to treatment with 3MC for 1 or 6 h. The protein levels of acetylated H3/H4, Cdk2/4, and Cyclin D3/E were assayed using Western blot. Membranes were probed using an anti-GAPDH antibody to verify equivalent loading. Three samples were analyzed in each group, and the values reported represent mean ± SEM (*P<0.05 vs. control group; ^#^ *P*<0.05 vs. 3MC treatment alone). (D) Cell numbers were counted for cells with indicated siRNA transfection followed by a 24 h of the 3MC challenge by using a hemo-cytometer. Data are presented as mean ± SEM of 3 independent experiments (*P<0.05 vs. control or siRNA group; ^#^ *P*<0.05 vs. 3MC treatment alone).

To verify the previous finding that 3MC activates RhoA through an AhR-dependent mechanism in HUVECs [3], we used CYP1A1, an AhR downstream effector, as a positive control to investigate the effect and AhR/RhoA action of 3MC in CMVECs and its underlying mechanisms. The concentration of 100 nM 3MC was chosen based on the concentration-dependent effects of 3MC in the induction of CYP1A1 (Figure S1A), and on the EC50 of 3MC observed in the DRE luciferase assay of MCVECs (Figure S1B). The MCVECs were transiently treated with 3MC at the indicated times with the evidence of CYP1A1 induction as the result of 3MC-mediated AhR activation (Figure 1A). 3MC significantly increased the nuclear content of AhR levels as CYP1A1 expression increased as early as 10 min after 3MC treatment was administered. The treatment with 3MC time-dependently reduced active forms of Ras and c-Raf in membrane fraction, and p-cRaf, whereas the MAPK family (Erk1/2, JNK, and p38) was not significantly altered. The alteration in Ras/c-Raf by 3MC was accompanied by a time-dependent inhibition of cell-cycle regulatory proteins (Figure 1). The affected genes and proteins included Cdk2/4 and Cyclin D3/E, and the observations were performed between 4 and 6 h after 3MC treatment (Figure 1B). In addition, the inactivation of Ras and c-Raf by 3MC was preceded by pRb2 hypophosphorylation and HDAC1 upregulation, but no significant alterations in HDAC2/4 levels. This led to H3/H4 deacetylation (Figure 2A). Immunofluorescent staining confirmed that the 3MC challenge increased the nuclear translocation of pRb2 and HDAC1 between 1 and 4 h after treatment (Figure 2B). The effect of HDAC1 activation on gene regulation of the cell-cycle regulatory proteins was investigated by applying siHDAC1, and the results were examined using Western blot analysis (Figure 2C). The results showed that siHDAC1 significantly reversed the 3MC-mediated H3/H4 deacetylation at 1 h in concomitant recovery in histone acetylation; furthermore, at 6 h after treatment, the protein levels of Cdk2/4 and Cyclin D3/E were rescued by the HDAC1 knockdown. In accordance with Pang et al., [4], we executed the experiments by using the loss-of-function HDAC1 approach to examine the functional significance of the downregulation of these cell-cycle regulatory proteins through epigenetic modulation in relation to the induction of p21/p27 by 3MC. In addition to the knockdown of p21/p27, the cells were simultaneously transfected with siHDAC1, and the prevention of cellular number reduction was compared with p21/p27-silencing cells. The results in Figure 2D demonstrate that cells transfected with siHDAC1 can increase cellular proliferation by approximately 33% in response to 24 h of the 3MC challenge. Additionally, the combined transfection of siHDAC1 with sip21/sip27 can further increase cell proliferation to a level comparable to the 24 h control group, suggesting that in addition to the effects from p21 and p27, HDAC1 plays an addictive role in recovering the reduced cell proliferation caused by the 3MC challenge.

### Effects of AhR and RhoA activation on pRb2/HDAC1 gene regulation and subcellular localization

**Figure 3:**
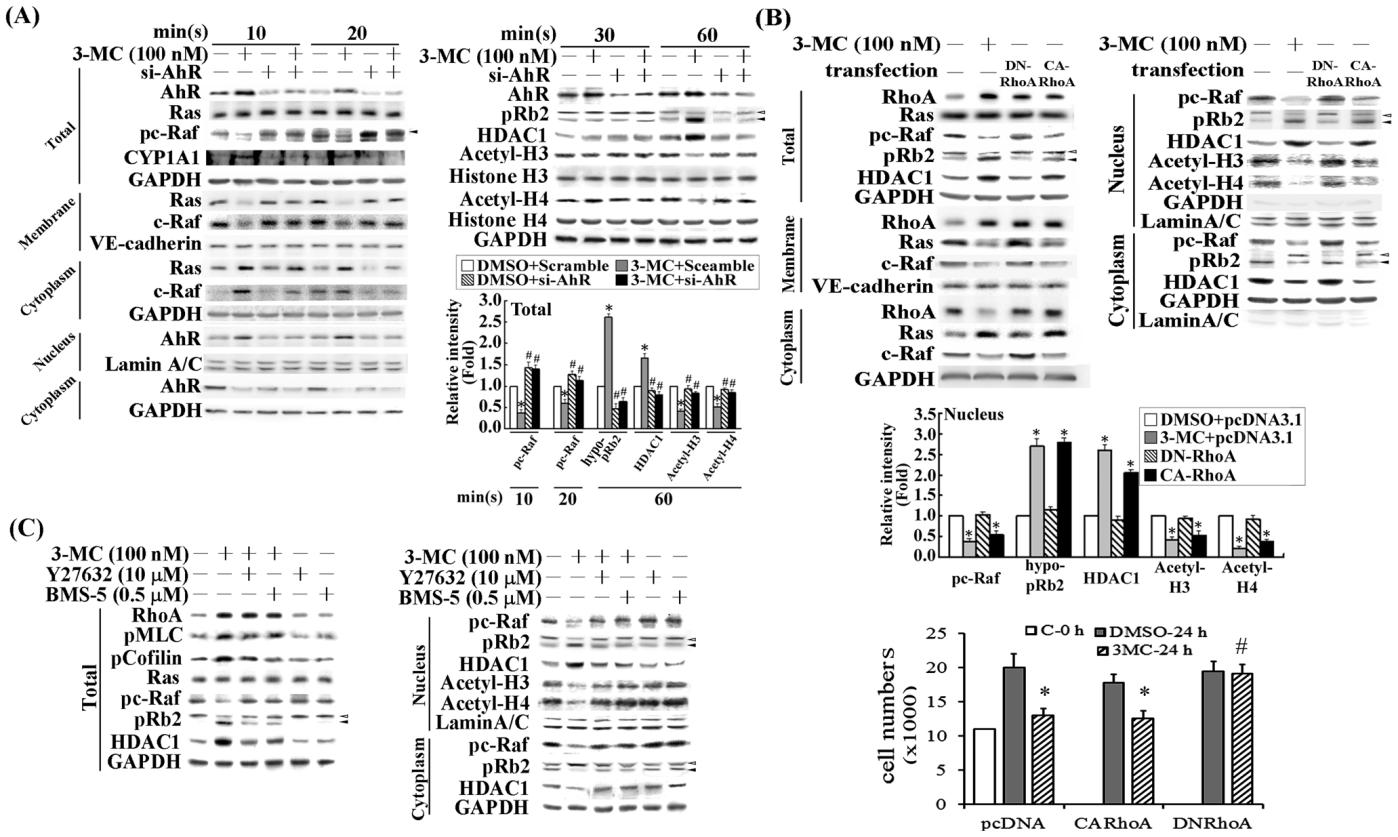
Essential role of AhR/RhoA activation in pRb2/HDAC1 upregulation and nuclear translocation induced by 3MC in MCVECs. Cells were transfected with siAhR (A), DNRhoA, or CARhoA (B) overnight, with or without 3MC treatment for 1 h and separated into membrane, cytosolic and nuclear fractions. Western blot analysis was used to examine Ras/c-Raf cytosolic-membrane distribution and AhR/pRb2/HDAC1 cytosolic-nuclear distribution by cellular fractionation. The effects of DNRhoA and CARhoA in cell proliferation were assessed by counting the cell numbers using a hemo-cytometer. Data are presented as mean ± SEM of 3 independent experiments. (C) Cells received a 1 h pretreatment with a ROCK inhibitor (Y27632) or a LIMK inhibitor (BMS-5), followed by 1 h of the 3MC challenge; the cells were then harvested and partitioned into nuclear and cytosolic fractions. Phosphorylation of myosin light chain and cofilin were used as positive controls for the actions of Y27632 and BMS-5, respectively. GAPDH, Lamin A/C, and VE-cadherin were used as internal controls for the cytosol, nuclear and membrane fractions, respectively, to verify equivalent loading. Representative results of 3 separate experiments are shown, and data are presented as the mean ± SEM (*P<0.05 vs. control or DMSO group; ^#^ *P*<0.05 vs. 3MC treatment alone).

Our previous studies have shown the key roles of AhR and RhoA in 3MC-mediated alterations of the vascular endothelial cells, including cellular proliferation, migration, the gene regulation of fibronectin, and α5β1 expression [3], [5]. In this study, we used the technique of gain- and loss-of-function of AhR and RhoA to examine the role of RhoA in pRb2/HDAC1 gene regulation, and subcellular localization. Cells displaying AhR knockdown or the overexpression of CARhoA or DNRhoA were harvested and fractionated after the 3MC challenge. The results (Figure 3A) indicated that AhR-silencing cells reduced the active form of Ras in membrane and p-cRaf at 10 or 20 min after the 3MC challenge. This was followed by reversed levels of pRb hypophosphorylation and HDAC1; thereafter, at 60 min following treatment, H3/H4 acetylation was observed (Figure 3A). Prior studies have similarly shown that increased RhoA levels and activity caused NF-κB nuclear translocation. Therefore, we examined the effect of RhoA activation on pRb2/HDAC1 nuclear translocation in cells overexpressing CARhoA and DNRhoA, followed by cytosolic and nuclear fractionation. The results showed that cotransfection with constitutively active RhoA (CARhoA) mimicked the effect of 3MC in increasing the nuclear translocation of pRb2/HDAC1 (Figure 3B). By contrast, cotransfection with a dominant negative RhoA (DNRhoA) showed the opposite effects in reversing the nuclear levels of pc-Raf, hypophosphorylated pRb2, HDAC1 and the histone H3/H4 deacetylation caused by 3MC. Their functional impact in cellular proliferation was assessed by counting the number of cells. Cells overexpressing CARhoA mimicked the effect of 3MC in the reduction of cell numbers, whereas those with DNRhoA significantly alleviated the reduced cell proliferation caused by the 3MC challenge. We also investigated the involvement of RhoA downstream effectors (ROCK and LIMK) in the 3MC-mediated increased nuclear translocation of pRb2/HDAC1; for this assessment we used Y27632 and BMS-5, inhibitors of ROCK and LIMK respectively. The results (Figure 3C) show that Y27632 and LIMK inhibitors can partially prevent 3MC-mediated increases in pRb2/HDAC1 nuclear translocation, implying a crucial role of RhoA activation in this event.

### Essential roles of PTEN, PI3K and Ras in 3MC-mediated chromatin deacetylation through a c-Raf and pRb2-dependent pathway

**Figure 4:**
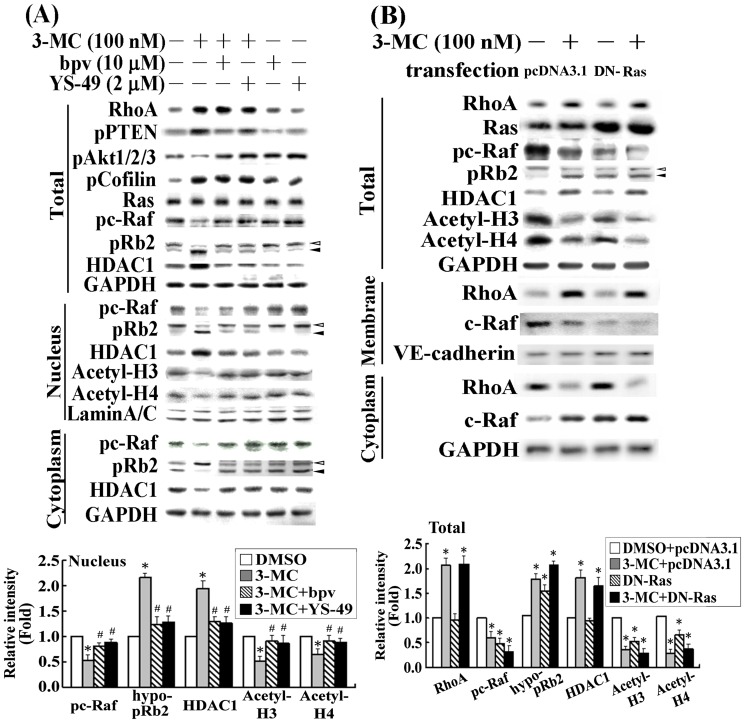
Involvement of PTEN and PI3K in pRb2 hypophosphorylation induced by Ras/c-Raf inactivation. (A) Cells were pretreated with bpv (an inhibitor of PTEN) for 30 min or YS-49 (an activator of PI3K) for 1 h, followed by 1 h of 100 nM 3MC treatment. Total cell lysates, and extraction of nuclear and cytosolic proteins were analyzed using Western blot analysis for related signaling molecules involved in chromatin deaceylation. (B) Cells were transfected with a plasmid containing the DNRas gene to mimic the effect of 3MC. After 1 h of 3MC treatment, cells were harvested for Western blot analysis of the mentioned molecules in total and cytosolic-membrane extracts. GAPDH, Lamin A/C, and VE-cadherin were used as internal controls for the cytosol (or total), nuclear and membrane fractions, respectively, to verify equivalent loading. The lower panel shows the intensity of bands in the Western blots using densitometry. Data are presented as mean ± SEM of 3 independent experiments (*P<0.05 vs. control group; ^#^ *P*<0.05 vs. 3MC treatment alone).

We previously demonstrated that 3MC treatment increased PTEN activity, which in turn decreased PI3K activity [5]. In the current study we examined the effect of 3MC-mediated alteration in the PTEN/PI3K pathway for pRb2 hypophosphorylation. We used the PTEN inhibitor bpv and the PI3K activator YS-49 for this assessment. The results (Figure 4A) show that in cells treated with bpv or YS-49, the 3MC-mediated Ras and c-Raf inactivation were reversed. This suggested that Ras and c-Raf inactivation were regulated by both PTEN (positive) and PI3K (negative). We then transfected cells with dominant negative Ras (DNRas) to examine whether the inactivation of Ras would mimic the effect of the 3MC on c-Raf dephosphorylation, thus resulting in pRb2 hypophosphorylation. Cells overexpressing DNRas displayed an effect similar to the 3MC in histone H3/H4 deacetylation, in a manner that depended on c-Raf/pRb2; this finding suggests that Ras plays an essential role in such regulation (Figure 4B).

### Increased HDAC1-E2F1 complex formation and the decreased E2F-driven transactivation by 3MC thereafter

**Figure 5:**
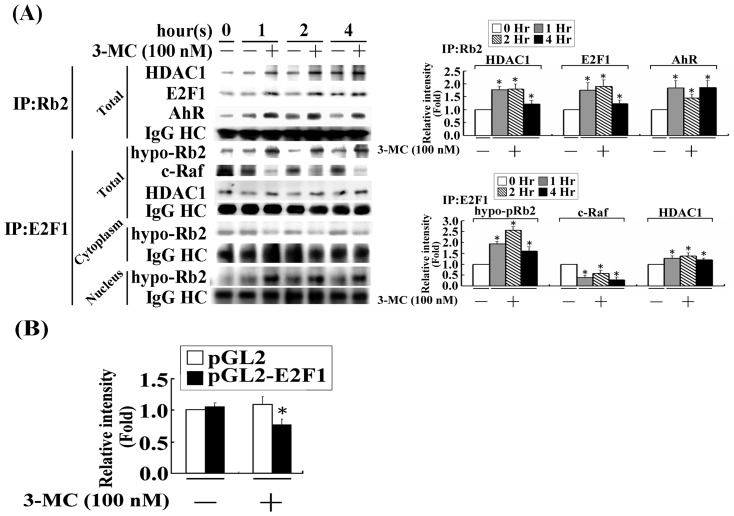
3MC treatment of MCVECs increased the formation of AhR/c-Raf/pRb2/E2F1/HDAC1 complexes, with a concomitant decrease in E2F-driven luciferase activity. (A) Cells received 1 h of 100 nM 3MC treatment; they were then harvested with subcellular fractionation and immune-precipitated by anti-pRb2 and anti-E2F1 antibodies (2 μg) and by protein A and G agarose beads (20 μL). The resulting immunoprecipitate was detected with anti-AhR, -E2F1, -c-Raf, -HDAC1, and - pRb2 antibodies in Western blot. The samples were normalized to the IgG heavy chain, the corresponding no 3MC signal, and the 0 h time point. (B) MCVECs were transiently transfected with pGL2/E2F and pRL-TK for 24 h, as described in the “Materials and Methods” section. Luciferase activity of the reported plasmid was normalized to that of the internal control plasmid. Data were derived from 3 independent experiments and are presented as mean ± SEM (*P<0.05 vs. the control group).

A co-immunoprecipitation assay was employed to examine whether the increased nuclear translocation would cause pRb2 and HDAC1 (Figure 2B) to interact with each other or participate in the E2F1 complex formation. The Co-IP results from using an anti-pRb2 antibody showed that the 3MC challenge not only increased the interaction between pRb2 and HDAC1, but also enhanced the AhR recruitment to this complex. In addition, a Co-IP assay using an anti-E2F1 antibody (Figure 5A) showed that E2F1 was part of the pRb2-HDAC1 complex; the interaction of this complex with c-Raf was decreased by the 3MC treatment of the MCVECs. These finding suggest that c-Raf participates in pRb2 phosphorylation. To dissect protein interaction in different cell compartments, cells with cytosolic-nuclear fractionation were immune-precipitated with anti-E2F1 antibody for analyzing its interaction with Rb2. After cytosolic and nuclear fractionation of cell lysate, the results in Figure 5A showed augmented E2F1-pRb2 interaction upon 3MC challenge in nuclear fraction, but reduced interaction in cytosolic fraction. Additionally, we observed that only a single band of pRb2 was found in the immune-precipitates using an anti-E2F1 antibody, which is a hypo-phosphorylated form of pRb2, based on its molecular weight. No any hyper-phosphorylated pRb2 protein band was observed in immunoprecipitates of cytosol and nuclear extracts of cell lysates with E2F antibody, either treated or untreated with 3MC. Moreover, we examined the effect of a 3MC-mediated increase on HDAC1 recruitment to the E2F1 complex in E2F transactivational activity using an E2F-driven luciferase reporter. The results (Figure 5B) show that 3MC significantly decreased E2F transactivational activity, compared with the control group.

### Alleviation of the increasing binding activities of E2F1 and HDAC1 to the promoters of cell-cycle regulatory proteins in 3MC-treated MCVECs by simvastatin

**Figure 6:**
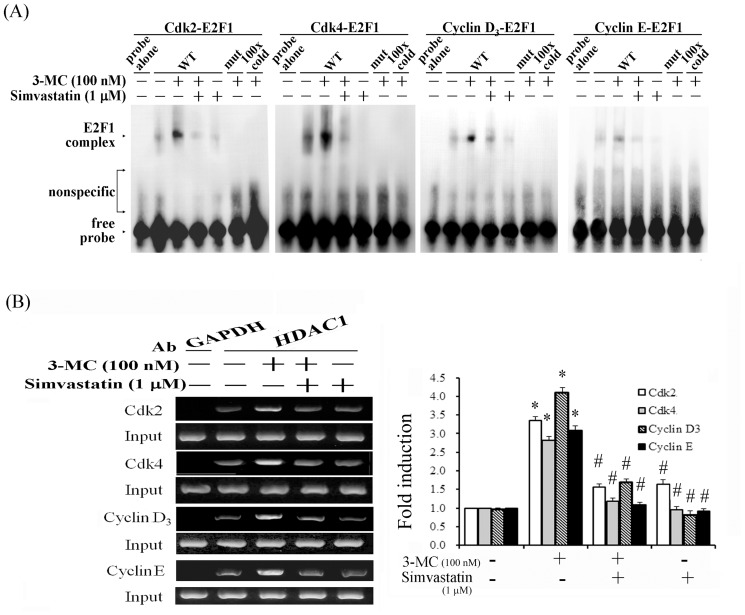
Elimination of 3MC-mediated increases in E2F/HDAC1 binding to the E2F responsive element in the promoters of Cdk2/4 and CyclinD3/E1 by simvastatin treatment. (A) Cells were cultured and treated with 100 nM of 3MC for 1 h after 1 h of simvastatin pretreatment. Nuclear proteins were assayed for E2F binding activity by WT and mut probes in an EMSA assay, as described in Materials and Methods. The term “100xcold” denotes a 100-fold molar excess of unlabeled oligonucleotides relative to the biotin-labeled probe; this was added to the binding assay to compete with the unlabeled oligonucleotides. The mobility of the E2F-E2F responsive element complex is indicated. Representative results of 3 experiments are shown. (B) A ChIP assay was performed in cells that received simvastatin pretreatment for 1 h, or followed by the 3MC challenge for 1 h, as indicated. The DNA associated with HDAC1 was immunoprecipitated with an anti-HDAC1 antibody; thereafter, PCR amplification was used to determine the extent of the association between HDAC1 and the functional E2F-binding sites in the promoters of Cdk2/4 and Cyclin D3/E1. An anti-GAPDH antibody was used as a negative control for the ChIP assays. Representative results of 3 experiments are shown, and data are presented as the mean ± SEM (*P<0.05 vs. the control; ^#^ *P*<0.05 vs. 3MC treatment alone).

To determine the effect of 3MC on the binding of E2F1 and HDAC1 to E2F-RE in the promoter regions of Cdk2/4 and Cyclin D3/E, we performed an EMSA and a chromatin IP assay. Based on the silico analysis using MatInspector professional software, putative E2F-REs in these target genes were identified at the following positions: −369 bp to −353 bp for Cdk2, −373 bp to −357 bp for Cdk4, −1660 bp to −1644 bp for Cyclin D3, and −1233 bp to −1217 bp for Cyclin E. The results (Figure 6A) using putative E2F-RE derived from their promoter regions show that 3MC increased the DNA-binding activities of E2F1 to E2F-RE. In light of the dependence of RhoA activation in augmenting nuclear translocation of pRb2/HDAC1 to form an E2F1 complex in MCVECs (Figure 3), we examined whether treatment with simvastatin, which is an inhibitor of RhoA activation, would prevent 3MC-mediated increases in the binding activities of E2F1 with the promoters of the target genes. The binding activities were abolished by simvastatin, their respective mutants, and competition from a 100-fold molar excess of unlabeled oligonucleotides (relative to the biotin-labeled probe). Furthermore, we examined the effects of 3MC on the association of HDAC1 with E2F1-REs in their target promoters, using a ChIP assay. We caused a 3MC-induced association between HDAC1 and the E2F1-responsive elements by using an anti-HDAC1 antibody to pull down the target fragments. The immunoprecipitated E2F-binding site fragments were amplified by RT-PCR to examine the association using primers derived from the target promoters. The ChIP assay (Figure 6B) showed that 3MC increased the levels of enrichment of the promoters of Cdk2/4 and Cyclin D3/E at 1 h of treatment, which were alleviated by an additional treatment with simvastatin.

### Statin derivatives rescued 3MC-mediated downregulation of Cdk2/4 and Cyclin D3/E and BrdU incorporation by RhoA inactivation

**Figure 7:**
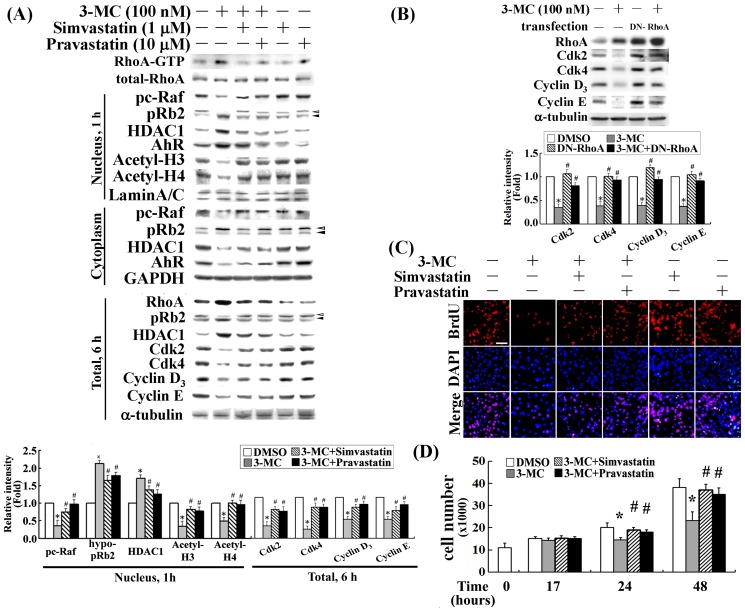
Effect of statins in preventing a 3MC-mediated decrease in cell-cycle regulatory proteins and in DNA incorporation induced by RhoA inactivation. Cells were pretreated with simvastatin or pravastatin for 1-cytosol and nuclear-cytosol of 3MC-treated MCVECs was analyzed to determine the action of statins in RhoA inactivation and their effect in preventing 3MC-mediated alterations in c-Raf/pRb2/HDAC1/histone deacetylation. (A) We analyzed fractionation after 1 h; thereafter, the levels of cell-cycle regulatory proteins were assessed by Western blot after 4 or 6 h of 3MC treatment. (B) Cells were transfected overnight with a plasmid containing DNRhoA, and 3MC treatment was administered for 6 h. A western blot analysis was conducted to examine the effect of DNRhoA on the cell-cycle regulatory proteins reduced by 3MC. GAPDH (or α-tubulin), Lamin A/C, and VE-cadherin were used as internal controls for the cytosol (or total), nuclear and membrane fractions, respectively, to verify equivalent loading. (C) Cells were pretreated with statins for 1 h; this was followed by the 3MC challenge, which was pulsed for 15 h with BrdU (Invitrogen; 0.75 μg/mL) incubation for the DNA incorporation assay. Fixed cells on coverslips were stained with a mouse anti-BrdU antibody conjugated with Texas Red. Red represents BrdU-positive staining. Identical fields were stained with DAPI (Invitrogen) to reveal the positions of cell nuclei. We recorded micrographs of the representative fields at 200× magnification (scale bar in white  = 250 μm). (D) Cell numbers were counted using a hemo-cytometer at the indicated time points in cells with various treatments. Data are presented as mean ± SEM of 3 independent experiments (*P<0.05 vs. control group; ^#^ *P*<0.05 vs. 3MC treatment alone).

Our findings show that 3MC-mediated RhoA activation was essential for the epigenetic modification of histones through a pRb2/HDAC1-depedent pathway. We examined whether treatment with simvastatin and pravastatin would prevent a 3MC-mediated decreased expression in cell-cycle regulatory proteins using Western blot analysis. The results (Figure 7A) show that simvastatin and pravastatin significantly decreased the activated form of RhoA, RhoA-GTP, and then eliminated the nuclear translocation of pRb2 and HDAC1 in a c-Raf-dependent fashion; this resulted in the recovery of histone H3/H4 acetylation. The results also indicate that statin derivatives rescued 3MC-mediated decreases in Cdk2/4 and Cyclin D3/E induction. In addition and unexpectedly, simvastatin or pravastatin decreased the levels of pRb2/HDAC1. This observation might be attributable to the reduction in AhR nuclear translocation (Figure 7A) and thereafter its downstream target gene transactivation. In agreement with the prevention of 3MC-mediated downregulation of Cyclin D3/E and Cdk2/4 by statins, the inactivation of RhoA in cells overexpressing DNRhoA significantly restored the levels of cell-cycle regulatory proteins reduced by 3MC, as shown in Figure 7B. Additionally, one of our previous studies showed that 3MC caused cell-cycle arrest in the G0/G1 phase. Herein, BrdU incorporation and cell counting assays were used to examine the potential of simvastatin and pravastatin in preventing 3MC-mediated anti-proliferation. As shown in Figure 7C, the inhibition of DNA incorporation by 3MC was significantly rescued by pretreatment with either simvastatin or pravastatin. However, we did not observe significant cell death using a TUNEL assay after 17 h of 3MC treatment (data not shown), suggesting that cell death might not contribute to the significant reduction in DNA synthesis. Significant cell death was not observed until 24 h of 3MC treatment (data not shown). Figure 7D shows that a significant reduction in cell number was apparent at 24–48 h, but not at 17 h of the 3MC challenge. The reduced cell numbers caused by 3MC was significantly rescued by pretreatment with either simvastatin or pravastatin; this finding suggests the critical role of RhoA in 3MC-mediated reduction of cellular proliferation.

## Discussion

**Figure 8:**
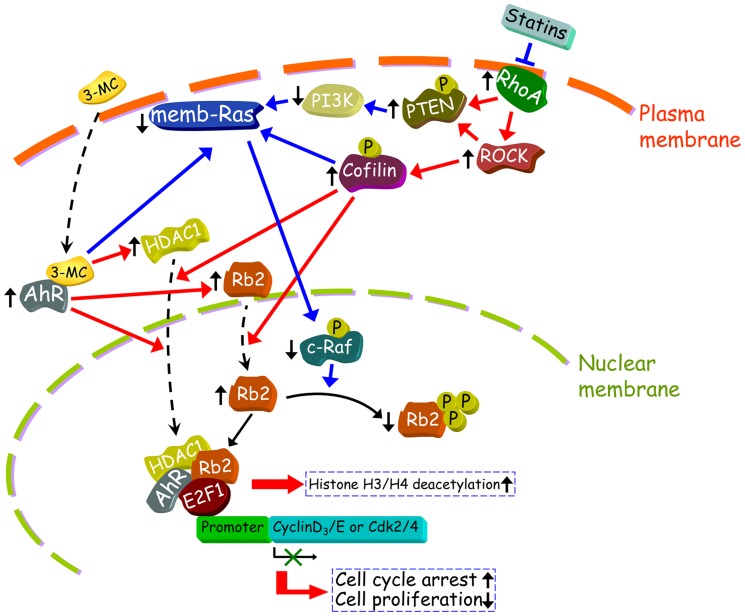
Summary of the signal pathways of 3MC on epigenetic modification of chromatin, resulting in MCVEC cell-cycle arrest. In the proposed signal pathways, 3MC-mediated AhR/RhoA activation modulates the PTEN/PI3K/Ras/c-Raf signaling pathways; this is followed by pRb2 hypophosphorylation, with a concomitant increase in HDAC1 upregulation and nuclear translocation. Treatment with 3MC also increased protein interaction within the E2F1 complex, including AhR, pRb2, and HDAC1. The downstream transcription factor E2F1 was negatively regulated by the co-repressor HDAC1, resulting in the downregulation of the cell-cycle regulatory proteins, including Cdk2/4 and Cyclin D3/E. Subsequently, DNA incorporation was decreased in MCVECs. The orange arrow lines indicate upregulation or activation of downstream molecules, whereas the blue lines represent downregulation or inactivation of the downstream targets. The signal pathways identified in this study are shown as solid lines with arrows, and proposed correlations are indicated by dashed lines with arrows. Additionally, the dashed-black lines indicate that the translocation occurs between cytosol and nuclei or between cytosol and the plasma membrane.

Our previous study using HUVECs showed that treatment with a 3MC arrested the cell cycle in the G0/G1 phase and exerted antiangiogenic and antiadhesive effects [27]. We subsequently showed that p21 and p27 were associated with the antiproliferative effects of 3MC in HUVECs, with induction occurring through functional DRE enhancers in the promoter regions of p21 and p27 [4]. Herein, we showed that 3MC-mediated AhR/RhoA activation was responsible for the epigenetic modification of chromatin in the gene regulation of the cell-cycle regulatory proteins in MCVECs. This modification resulted in decreased DNA incorporation and proliferation in the cells treated with 3MC (as summarized in Figure 8).

In the current study, we examined the effects of 3MC treatment on MCVECs. The results show that the AhR/RhoA activation induced by 3MC increased pRb2/HDAC1 upregulation and pRb2 hypophosphorylation, with the hypophosphorylation occurring through a PTEN/PI3K/Ras/c-Raf-dependent pathway. Inductions of pRb2/HDAC1 were then translocated into nuclei to form a complex with E2F1, which caused the downregulation of cell-cycle regulatory proteins, including Cdk2/4 and Cyclin D3/E. Thereafter, DNA incorporation was decreased (Figure 7C). Further evidence using the gain- and loss-of-function of AhR/RhoA and pharmaceutical inhibitors of the RhoA downstream targets ROCK and LIMK showed that RhoA is responsible for the nuclear translocation of pRb2/HDAC1 normally induced by 3MC (Figure 3). A recent study reported similar findings in HUVECs [22], which showed that cofilin-1 (a downstream target of RhoA/ROCK/LIMK1) played an essential role in regulating thrombin-induced relA/p65 nuclear translocation and intercellular adhesion molecule 1 (ICAM-1) expression.

Our findings differed from studies claiming that the pathway of Ras-cRaf-Erk is activated by AhR agonists [28], [29]. Our results showed that in MCVECs treated with 3MC, Ras/c-Raf was inactivated through an AhR/RhoA-dependent mechanism (Figure 3) and that PTEN/PI3K was involved in the dephosphorylation of c-Raf/pRb2 in MCVECs (Figure 4A). In agreement with our findings, several reports showed the requirement of pRb in the PTEN-induced inhibition of cell proliferation, because PTEN is unable to cause cell growth arrest in retinoblastoma (pRb)-deficient cell lines [30], [31]. Additionally, PTEN-induced growth arrest through inhibiting Ras activation [32] can be rescued by expressing constitutive active PI3K and downstream effectors such as Akt [33].

Furthermore, the Co-IP results showed that as well as enhancing HDAC1 recruitment, 3MC increased the binding of AhR and pRb2 to the E2F1 complex (Figure 5B). By contrast, 3MC inhibited the binding of c-Raf to this complex. These findings suggest that the pRb2 hypophosphorylation might result from the decreased interaction between pRb2 and c-Raf. The results of a recent study agreed that the disruption of the binding of c-Raf to pRb prevents pRb phosphorylation, cell cycle progression, and angiogenic tubule formation [34]. Moreover, our data also suggests that 3MC-liganded AhR physically interacted with the E2F1 complex in the downregulation of the cell-cycle regulatory genes. Our findings were congruent with those reported by Gomez-Duran et al., [35], in which the AhR increased the recruitment of HDAC1, 2 and 4, and this increased recruitment was correlated with the decreased K8H4 acetylation and impaired expression of latent TGFbeta-binding protein 1 [35]. Our results similarly show that a series of signal transduction molecules activated by AhR/RhoA in the increased recruitment of HDAC1 and pRb2 to the E2F1 complex (Figure 8) led to the downregulation of cell-cycle-regulatory proteins and thereafter reduced the DNA incorporation and cellular proliferation.

Eleven members of the HDAC family have been identified and designated HDAC1 to HDAC11. HDAC1 has been shown to act as a substrate for E2F1 [36] and contains a relatively high consensus sequence of the AhR-responsive element in its promoter region. In addition to the AhR binding sites found in HDAC1, our analysis (using MatInspector professional software) showed that the promoters of pRb2 also contained AhR binding sites (data not shown). This might explain the observation that the levels of HDAC1 and pRb2 were all subject to regulation by 3MC-mediated AhR activation. Unexpectedly, our results showed that RhoA inhibitors (simvastatin and pravastatin) also reversed the increased levels of HDAC1 and pRb2 induced by 3MC (Figure 7B). We reasoned that RhoA inactivation decreased the nuclear level of AhR, which in turn decreased the levels of pRb2 and HDAC1 caused by 3MC (Figure 7). Ours is the first study to show this phenomenon. However, we could not rule out the possibility that these statin derivatives might have exerted another unidentified effect in gene regulation. The underlying mechanisms require further investigation.

We previously showed that the induction of p21/p27 by 3MC occurred through AhR-dependent transcriptional regulation, resulting in cell-cycle arrest in HUVECs [4]. Similarly, the induction of p21/p27 was observed in MCVECs (data not shown). Furthermore, the induction of pRb2 that we observed in MCVECs was also evident in HUVECs. Thus, cells obtained from different species and tissues responded similarly to the 3MC challenge. This finding emphasizes the potency of environmental hazards that ubiquitously affect numerous biological organisms and tissues. In summary, our study shows that the epigenetic modification effect of 3MC on cell-cycle regulatory proteins occurs through increased recruitment of HDAC1 and pRb2 to the E2F1-DNA complex. We also identified functional E2F-binding sites located at the promoters of Cdk2/4 and Cyclin D3/E, which were negatively regulated through the HDAC1-E2F1 complex by 3MC-mediated RhoA activation.

This study and our previous studies have contributed to the understanding of 3MC-mediated inhibition in cell-cycle arrest in MCVECs; that is, the genomic upregulation of p21/p27, and the histone deacetylation involved in the downregulation of Cdk2/4 and Cyclin D3/E through AhR/RhoA-dependent mechanisms. This understanding of the molecular mechanisms of 3MC-mediated cell-cycle arrest suggests the possibility of using simvastatin or pravastatin to inhibit RhoA activation as a therapeutic approach to AhR agonist-mediated anti-proliferation in VECs. The decrease in cell proliferation of MCVECs might indicate a toxic effect from environmental pollutants or hormones such as 3MC, benzo[a]pyrene, and TCDD, and such substances might be harmful to the brain during hypoxia. Our findings provide insight into the potential molecular mechanisms of the action of 3MC in cerebral vascular injuries, and the use of simvastatin and pravastatin as a therapeutic intervention to reverse the process.

## Supporting Information

Figure S1 **The concentration-dependent effect of 3MC in CYP1A1 induction, an AhR downstream target, and the DRE-driven luciferase activity.** (A) MCVECs were treated with an increasing concentration of 3MC from 10 nM to 10 μM for 1 h. The resulting lysates were analyzed to determine the expression levels of the proteins of interest. (B) Cells were transiently transfected with pGL2/3DRE and pRL-TK for 24 h, as described in the Materials and Methods section. Cells were pretreated with the indicated concentrations of 3MC for 1 h. Luciferase activities demonstrated by the reported plasmid were normalized to those of the internal control plasmid. Data were derived from 3 independent experiments.
(TIF)

## References

[1] Ma Q, Whitlock JP Jr (1997) A novel cytoplasmic protein that interacts with the Ah receptor, contains tetratricopeptide repeat motifs, and augments the transcriptional response to 2,3,7,8-tetrachlorodibenzo-p-dioxin. J Biol Chem 272: 8878–8884. 9083006

[2] Denison MS, Whitlock JP Jr (1995) Xenobiotic-inducible transcription of cytochrome P450 genes. J Biol Chem 270: 18175–18178. 7629130 10.1074/jbc.270.31.18175

[3] Chang CC, Tsai SY, Lin H, Li HF, Lee YH, et al (2009) Aryl-hydrocarbon receptor-dependent alteration of FAK/RhoA in the inhibition of HUVEC motility by 3-methylcholanthrene. Cell Mol Life Sci 66: 3193–3205. 19649566 10.1007/s00018-009-0102-7 PMC11115561

[4] Pang PH, Lin YH, Lee YH, Hou HH, Hsu SP, et al (2008) Molecular mechanisms of p21 and p27 induction by 3-methylcholanthrene, an aryl-hydrocarbon receptor agonist, involved in antiproliferation of human umbilical vascular endothelial cells. J Cell Physiol 215: 161–171. 18022818 10.1002/jcp.21299

[5] Chang CC, Lee PS, Chou Y, Hwang LL, Juan SH (2012) Mediating effects of aryl-hydrocarbon receptor and RhoA in altering brain vascular integrity: the therapeutic potential of statins. Am J Pathol 181: 211–221. 22720799 10.1016/j.ajpath.2012.03.032

[6] Lees E (1995) Cyclin dependent kinase regulation. Curr Opin Cell Biol 7: 773–780. 8608007 10.1016/0955-0674(95)80060-3

[7] Sherr CJ (1993) Mammalian G1 cyclins. Cell 73: 1059–1065. 8513492 10.1016/0092-8674(93)90636-5

[8] Sherr CJ, Roberts JM (1999) CDK inhibitors: positive and negative regulators of G1-phase progression. Genes Dev 13: 1501–1512. 10385618 10.1101/gad.13.12.1501

[9] Wang S, Ghosh RN, Chellappan SP (1998) Raf-1 physically interacts with Rb and regulates its function: a link between mitogenic signaling and cell cycle regulation. Mol Cell Biol 18: 7487–7498. 9819434 10.1128/mcb.18.12.7487 PMC109329

[10] DeGregori J (2006) Surprising dependency for retinoblastoma protein in ras-mediated tumorigenesis. Mol Cell Biol 26: 1165–1169. 16449632 10.1128/MCB.26.4.1165-1169.2006 PMC1367209

[11] Dudler T, Gelb MH (1996) Palmitoylation of Ha-Ras facilitates membrane binding, activation of downstream effectors, and meiotic maturation in Xenopus oocytes. J Biol Chem 271: 11541–11547. 8626715 10.1074/jbc.271.19.11541

[12] Claudio PP, Stiegler P, Howard CM, Bellan C, Minimo C, et al (2001) RB2/p130 gene-enhanced expression down-regulates vascular endothelial growth factor expression and inhibits angiogenesis in vivo. Cancer Res 61: 462–468. 11212232

[13] Garrison PM, Rogers JM, Brackney WR, Denison MS (2000) Effects of histone deacetylase inhibitors on the Ah receptor gene promoter. Arch Biochem Biophys 374: 161–171. 10666294 10.1006/abbi.1999.1620

[14] Latchney SE, Lioy DT, Henry EC, Gasiewicz TA, Strathmann FG, et al (2011) Neural precursor cell proliferation is disrupted through activation of the aryl hydrocarbon receptor by 2,3,7,8-tetrachlorodibenzo-p-dioxin. Stem Cells Dev 20: 313–326. 20486776 10.1089/scd.2009.0529 PMC3128757

[15] Puga A (2011) Perspectives on the potential involvement of the AH receptor-dioxin axis in cardiovascular disease. Toxicol Sci 120: 256–261. 21205634 10.1093/toxsci/kfq393 PMC3107491

[16] Ge NL, Elferink CJ (1998) A direct interaction between the aryl hydrocarbon receptor and retinoblastoma protein. Linking dioxin signaling to the cell cycle. J Biol Chem 273: 22708–22713. 9712901 10.1074/jbc.273.35.22708

[17] Puga A, Barnes SJ, Dalton TP, Chang C, Knudsen ES, et al (2000) Aromatic hydrocarbon receptor interaction with the retinoblastoma protein potentiates repression of E2F-dependent transcription and cell cycle arrest. J Biol Chem 275: 2943–2950. 10644764 10.1074/jbc.275.4.2943

[18] Weiss C, Kolluri SK, Kiefer F, Gottlicher M (1996) Complementation of Ah receptor deficiency in hepatoma cells: negative feedback regulation and cell cycle control by the Ah receptor. Exp Cell Res 226: 154–163. 8660951 10.1006/excr.1996.0214

[19] Elferink CJ, Ge NL, Levine A (2001) Maximal aryl hydrocarbon receptor activity depends on an interaction with the retinoblastoma protein. Mol Pharmacol 59: 664–673. 11259609 10.1124/mol.59.4.664

[20] Zhang HS, Dean DC (2001) Rb-mediated chromatin structure regulation and transcriptional repression. Oncogene 20: 3134–3138. 11420730 10.1038/sj.onc.1204338

[21] Nobes CD, Hall A (1999) Rho GTPases control polarity, protrusion, and adhesion during cell movement. J Cell Biol 144: 1235–1244. 10087266 10.1083/jcb.144.6.1235 PMC2150589

[22] Fazal F, Bijli KM, Minhajuddin M, Rein T, Finkelstein JN, et al (2009) Essential role of cofilin-1 in regulating thrombin-induced RelA/p65 nuclear translocation and intercellular adhesion molecule 1 (ICAM-1) expression in endothelial cells. J Biol Chem 284: 21047–21056. 19483084 10.1074/jbc.M109.016444 PMC2742869

[23] Xu J, He L, Ahmed SH, Chen SW, Goldberg MP, et al (2000) Oxygen-glucose deprivation induces inducible nitric oxide synthase and nitrotyrosine expression in cerebral endothelial cells. Stroke 31: 1744–1751. 10884482 10.1161/01.str.31.7.1744

[24] Lin H, Lee JL, Hou HH, Chung CP, Hsu SP, et al (2008) Molecular mechanisms of the antiproliferative effect of beraprost, a prostacyclin agonist, in murine vascular smooth muscle cells. J Cell Physiol 214: 434–441. 17620284 10.1002/jcp.21214

[25] Lam EW, Bennett JD, Watson RJ (1995) Cell-cycle regulation of human B-myb transcription. Gene 160: 277–281. 7642110 10.1016/0378-1119(95)00184-8

[26] Shih CM, Lin H, Liang YC, Lee WS, Bi WF, et al (2004) Concentration-dependent differential effects of quercetin on rat aortic smooth muscle cells. Eur J Pharmacol 496: 41–48. 15288573 10.1016/j.ejphar.2004.06.016

[27] Juan SH, Lee JL, Ho PY, Lee YH, Lee WS (2006) Antiproliferative and antiangiogenic effects of 3-methylcholanthrene, an aryl-hydrocarbon receptor agonist, in human umbilical vascular endothelial cells. Eur J Pharmacol 530: 1–8. 16359657 10.1016/j.ejphar.2005.11.023

[28] Pierre S, Bats AS, Chevallier A, Bui LC, Ambolet-Camoit A, et al (2011) Induction of the Ras activator Son of Sevenless 1 by environmental pollutants mediates their effects on cellular proliferation. Biochem Pharmacol 81: 304–313. 20950586 10.1016/j.bcp.2010.10.003

[29] Mulero-Navarro S, Pozo-Guisado E, Perez-Mancera PA, Alvarez-Barrientos A, Catalina-Fernandez I, et al (2005) Immortalized mouse mammary fibroblasts lacking dioxin receptor have impaired tumorigenicity in a subcutaneous mouse xenograft model. J Biol Chem 280: 28731–28741. 15946950 10.1074/jbc.M504538200

[30] Brennan P, Babbage JW, Burgering BM, Groner B, Reif K, et al (1997) Phosphatidylinositol 3-kinase couples the interleukin-2 receptor to the cell cycle regulator E2F. Immunity 7: 679–689. 9390691 10.1016/s1074-7613(00)80388-x

[31] Klippel A, Escobedo MA, Wachowicz MS, Apell G, Brown TW, et al (1998) Activation of phosphatidylinositol 3-kinase is sufficient for cell cycle entry and promotes cellular changes characteristic of oncogenic transformation. Mol Cell Biol 18: 5699–5711. 9742087 10.1128/mcb.18.10.5699 PMC109156

[32] Gu J, Tamura M, Yamada KM (1998) Tumor suppressor PTEN inhibits integrin- and growth factor-mediated mitogen-activated protein (MAP) kinase signaling pathways. J Cell Biol 143: 1375–1383. 9832564 10.1083/jcb.143.5.1375 PMC2133067

[33] Paramio JM, Navarro M, Segrelles C, Gomez-Casero E, Jorcano JL (1999) PTEN tumour suppressor is linked to the cell cycle control through the retinoblastoma protein. Oncogene 18: 7462–7468. 10602505 10.1038/sj.onc.1203151

[34] Davis RK, Chellappan S (2008) Disrupting the Rb-Raf-1 interaction: a potential therapeutic target for cancer. Drug News Perspect 21: 331–335. 18836591 10.1358/dnp.2008.21.6.1246832 PMC2800199

[35] Gomez-Duran A, Ballestar E, Carvajal-Gonzalez JM, Marlowe JL, Puga A, et al (2008) Recruitment of CREB1 and histone deacetylase 2 (HDAC2) to the mouse Ltbp-1 promoter regulates its constitutive expression in a dioxin receptor-dependent manner. J Mol Biol 380: 1–16. 18508077 10.1016/j.jmb.2008.04.056 PMC2824431

[36] Martinez-Balbas MA, Bauer UM, Nielsen SJ, Brehm A, Kouzarides T (2000) Regulation of E2F1 activity by acetylation. EMBO J 19: 662–671. 10675335 10.1093/emboj/19.4.662 PMC305604

