# Supplementary figures and images for: 3-Methylcholanthrene, an AhR Agonist, Caused Cell-Cycle Arrest by Histone Deacetylation through a RhoA-Dependent Recruitment of HDAC1 and pRb2 to E2F1 Complex
Source: PLoS One. 2014 Mar 21;9(3):e92793. doi: 10.1371/journal.pone.0092793 (PMC3962457; doi:10.1371/journal.pone.0092793)

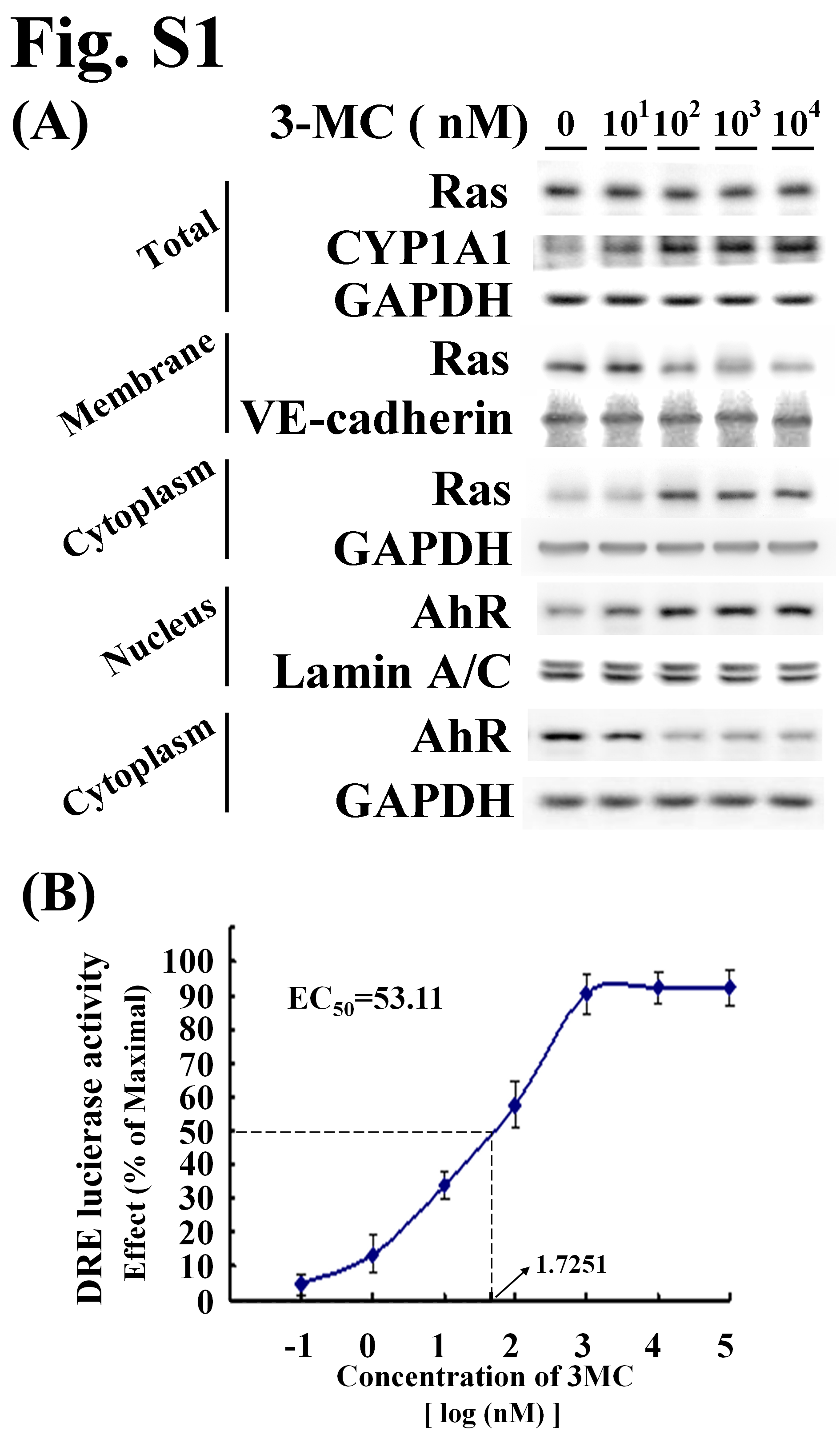

Supplement: Figure S1 — The concentration-dependent effect of 3MC in CYP1A1 induction, an AhR downstream target, and the DRE-driven luciferase activity. (A) MCVECs were treated with an increasing concentration of 3MC from 10 nM to 10 μM for 1 h. The resulting lysates were analyzed to determine the expression levels of the proteins of interest. (B) Cells were transiently transfected with pGL2/3DRE and pRL-TK for 24 h, as described in the Materials and Methods section. Cells were pretreated with the indicated concentrations of 3MC for 1 h. Luciferase activities demonstrated by the reported plasmid were normalized to those of the internal control plasmid. Data were derived from 3 independent experiments. (TIF) [file pone.0092793.s001.tif]
